# Treatment of Cytomegalovirus Infection with Cidofovir and CMV Immune Globulin in a Lung Transplant Recipient

**DOI:** 10.1155/2016/4560745

**Published:** 2016-01-13

**Authors:** Heinrike Wilkens, Martina Sester

**Affiliations:** ^1^Department of Internal Medicine V-Pneumology, Allergology, Respiratory and Environmental Medicine, University Hospital of Saarland, Kirrbergerstrasse 100, Homburg, 66421 Saarland, Germany; ^2^Department of Transplant and Infection Immunology, Saarland University, Kirrbergerstrasse, Building 47, Homburg, 66421 Saarland, Germany

## Abstract

Cytomegalovirus (CMV) infection after lung transplantation is associated with increased risk for pneumonitis and bronchiolitis obliterans as well as allograft rejection and opportunistic infections. Ganciclovir is the mainstay of prophylaxis and treatment but CMV infections can be unresponsive. Apart from direct antiviral drugs, CMV immunoglobulin (CMVIG) preparations may be considered but are only licensed for prophylaxis. A CMV-seronegative 42-year-old man with cystic fibrosis received a lung from a CMV-seropositive donor. Intravenous ganciclovir prophylaxis was delayed until day 12 due to acute postoperative renal failure and was accompanied by five doses of CMVIG (10 g). By day 16, CMV-DNA was detectable and rising; CMV-specific T-cells were undetectable. Switch from ganciclovir to foscarnet prompted a transient decrease in CMV viral load, but after increasing again to reach 3600 copies/mL foscarnet was changed to intravenous cidofovir and CMVIG was restarted. CMV load continued to fluctuate and declined slowly, whereas CMV-specific T-cells were detected five months later and increased thereafter. At last follow-up, the patient was in very good clinical condition with no evidence of bronchiolitis obliterans. No side effects of this treatment were observed. In this hard-to-treat case, the combination of cidofovir with off-label use of CMVIG contributed to a successful outcome.

## 1. Introduction

Cytomegalovirus (CMV) infection is an important clinical concern after all types of solid organ transplantation, but the highest rates of CMV infection and CMV disease are seen in lung and heart-lung transplant patients [1]. Tissue-invasive CMV disease can affect various organ systems to cause severe and frequently fatal symptoms, but in lung transplant recipients pneumonitis is the most feared complication. CMV infection or CMV pneumonitis also increases the risk of bronchiolitis obliterans syndrome (BOS) after lung transplantation [2, 3], coupled with a higher propensity to develop graft rejection and opportunistic infection [4].

The cornerstone of management for CMV infection after organ transplantation is treatment with intravenous (i.v.) ganciclovir and its prodrug valganciclovir, but viral clearance is not achieved in all patients [5–7], necessitating additional or alternative interventions to avoid or manage tissue-invasive CMV disease. Such situations can arise, for example, if ganciclovir-resistant infection occurs [8], if optimal doses of ganciclovir cannot be given due to hematological or other side effects, or if renal function is impaired due to the risk of ganciclovir-related nephrotoxicity [9, 10]. Typically, another antiviral agent, most frequently foscarnet or cidofovir (both used off-label in this setting), is introduced according to the sensitivity of the responsible strain of CMV although again a response is by no means certain and intolerance due to bone marrow and renal toxicity can again be dose-limiting or require discontinuation [11]. Thus, there are cases in which nonstandard treatment strategies must be considered if progression of potentially fatal CMV-related disease is to be prevented [12].

CMV immunoglobulin (CMVIG) preparations are licensed for the prophylaxis of CMV infection and disease and are used by approximately a third of lung transplant centers for prophylaxis in high-risk (CMV-negative recipient/CMV-positive donor [R−/D+]) transplant procedures [13], although well-designed studies to assess its effectiveness in the setting of lung transplantation are lacking. CMVIG acts in a complementary manner to antiviral therapy. While the passive CMV-specific immunity provided by CMVIG administration eliminates circulating CMV particles via opsonization and phagocytosis, antiviral agents block intracellular viral replication by inhibiting DNA polymerase. Antiviral drugs thus exert a direct effect on viral replication, whereas CMVIG may contribute to decreased entry of CMV into the cell. There is therefore a rationale to use CMVIG as adjunct therapy for CMV viremia or CMV disease which proves unresponsive to conventional antiviral monotherapy. International guidelines state that CMVIG therapy can be considered to treat severe cases of CMV disease such as pneumonitis but emphasize that more evidence is required [14, 15].

We describe here a difficult-to-treat case of CMV infection in a lung transplant recipient who did not show a sustained response after introduction of foscarnet, prompting switch to combined treatment based on cidofovir with CMVIG.

## 2. Case Report

A 42-year-old man with cystic fibrosis underwent lung transplantation on 1 June 2009 at the Saarland University Medical Center in Homburg, Germany. He had been on home ventilation via tracheostomy since February 2009 due to respiratory failure. The patient was CMV-seronegative and received a graft from a CMV-seropositive donor. Two 20 mg doses of basiliximab (Simulect, Novartis Pharma AG, Basel, Switzerland) were given on days 0 and 4 after transplant. Maintenance immunosuppression comprised tacrolimus (Prograf, Astellas Pharma Inc., Tokyo, Japan) with azathioprine and prednisolone. The patient did not experience any acute rejection during follow-up and no antirejection therapy was required.

**Figure 1:**
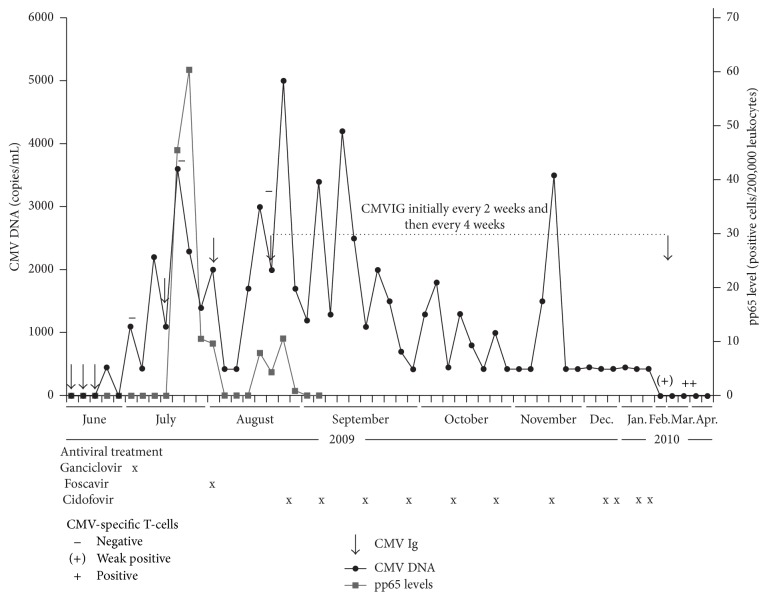
Schematic of CMV treatment, CMV-DNA, and pp65 levels over time. The absence and presence of CMV-specific CD4^+^ T-cells as determined by flow cytometry are indicated by (−) and (+) symbols. CMV-DNA was quantified from whole blood using the Cobas-Amplicor-assay (Roche Diagnostics) with a clinically relevant detection limit of 450 copies/mL. A cut-off of CMV-specific CD4^+^ T-cells of ≥0.05% was considered positive.

Two hours after transplantation, severe bleeding occurred. A rethoracotomy for bleeding control was necessary and the patient needed transfusions of thrombocytes, fresh frozen plasma, and seven erythrocyte concentrates. He developed acute postoperative renal failure requiring hemodialysis, which delayed the start of CMV prophylaxis. On 6 June (postoperative day 6), CMV-DNA and pp65 were negative (Figure 1). Two days later, hemodialysis could be stopped. Intravenous ganciclovir prophylaxis was started on 12 June (12 days after transplant). The dose of i.v. ganciclovir was adapted to renal function, at 2.5 mg/kg b.i.d. Five doses of CMVIG (10 g; Cytotect, Biotest AG, Dreieich, Germany) were also administered, on 1 June, 10 June, 18 June, 16 July, and 3 August. On 10 June, the patient was still negative for CMV-DNA and pp65, and he had no CMV-specific T-cells, which were assessed directly from whole blood after a 6-hour stimulation with a CMV lysate along with negative and positive control stimulations. Detection was based on intracellular IFN-*γ* staining in CD69^+^-positive activated CD4^+^ T-cells using flow cytometry, as described previously [16, 17]. By 16 June, however, CMV-DNA had become detectable (450 copies/mL), rising to 530 copies/mL two days later and to 1200 copies/mL by 23 June despite interruption of azathioprine therapy on 17 June to decrease the intensity of immunosuppression. At this point, he had not developed any CMV-specific T-cells, and there was concern that ganciclovir-related bone marrow toxicity may have suppressed their emergence. The decision was made to switch from ganciclovir to foscarnet (Foscavir, Clinigen, Burton-on-Trent, UK) on 23 June. There was a transient decrease in the CMV viral load after the start of foscarnet therapy, but by 23 July it had increased again to 3600 copies/mL (Figure 1). In response to this increase, on 21 August, foscarnet was discontinued and i.v. cidofovir (Vistide, Gilead Sciences Inc., Foster City, CA, USA) was started empirically at a dose of 5 mg/kg (294 mg), repeated on 28 August. Cidofovir administration was repeated at a dose of 5 mg/kg every two weeks from 11 September 2009 to 28 January 2010. In addition, CMVIG at a dose of 10 g was started on 24 August 2009 in response to the increasing CMV load, initially given every two weeks and then every four weeks until 1 March 2010. At that point, low levels of CMV-specific T-cells had been detected for the first time and increased thereafter. Triple immunosuppressive therapy was restarted. At last follow-up, in June 2015, the patient was in very good clinical condition with no evidence of bronchiolitis obliterans. Renal function was moderately impaired, with serum creatinine of 1.42 mg/dL and estimated GFR of 56 mL/min. The patient has remained negative for CMV-DNA and pp65 and highly positive for CMV-specific T-cells.

## 3. Discussion

The literature contains only a few case reports and no randomized trials regarding use of CMVIG to treat CMV infection after lung transplantation. In this patient who developed CMV infection approximately two weeks after lung transplantation, introduction of foscarnet, followed by switch to cidofovir combined with CMVIG, ultimately achieved viral clearance and a satisfactory clinical outcome. In this hard-to-treat case, CMVIG has off-label use as adjunctive therapy to antiviral drugs, being licensed only for the prophylaxis of CMV infection. The infection did not respond to ganciclovir or foscarnet, and CMV viral load was rising. Although ganciclovir resistance testing was not performed, the presence of resistance cannot be ruled out. A switch to cidofovir and continuing treatment with repeated doses of CMVIG at a relatively high dose led to the point where anti-CMV therapy could be withdrawn. No side effects of this treatment were observed.

The patient did not develop any CMV-specific T-cells during several months of CMV infection, although general T-cell function determined after polyclonal stimulation was readily detectable (data not shown). In this situation, the combined action of cidofovir and CMVIG was associated with a slow decrease in viral replication which may have eventually allowed induction of CMV-specific cellular immunity, which in turn may have contributed to control of viremia after stopping therapy. One may speculate that the combined treatment was effective, as CMVIG alone administered prophylactically did not prevent CMV primary infection in the first days after transplantation. The decision to stop treatment was guided by CMV-specific T-cell immunity as well as levels of CMV-DNA and pp65.

As demonstrated in this case, the induction of CMV-specific T-cell responses after primary infection can be used as a tool to assess the individual's ability to control virus replication in the absence of further therapy [16]. In CMV-seropositive transplant recipients, a decrease in the frequency of CMV-specific CD4^+^ T-cells may be predictive for CMV-associated disease [17, 18]. Individual levels of CMV-specific T-cells may decline due to increased viral replication and consumption of specific T-cells. Since an impaired CMV-specific T-cell response may help to identify patients at risk for uncontrolled viral replication, monitoring of CMV-specific CD4^+^ T-cells and viral load could support targeting of antiviral therapy and determination of the optimal duration [16, 17].

The case has several limitations. It could be speculated that starting CMV prophylaxis earlier in this high-risk patient with CMV serology mismatch might have avoided CMV primary infection and the need for extended treatment. The prolonged failure to mount an adequate CMV-specific T-cell response despite detectable CMV viremia, however, is associated with a poor prognosis and lack of response to CMV treatment. In addition, since cidofovir and CMVIG were coadministered, the individual contribution to control of viremia cannot be determined. In addition to the neutralizing effect of CMV-specific humoral immunity, the immunomodulatory effect of CMVIG treatment may have contributed to preservation of good lung function despite reduced immunosuppression, with the patient showing supranormal expiratory flow-volume curves. This type of treatment may therefore be considered as adjunct therapy in patients with a poor response to direct antiviral drugs such as ganciclovir or foscarnet. Management of CMV complications after lung transplantation may be further improved by monitoring of both CMV-specific CD4^+^ T-cells and viral load to guide the intensity and duration of CMV treatment, with combined administration of CMVIG and antiviral drugs during phases of viral replication.
